# Dyslipidemias and Elevated Cardiovascular Risk on Lopinavir-Based Antiretroviral Therapy in Cambodia

**DOI:** 10.1371/journal.pone.0160306

**Published:** 2016-08-31

**Authors:** Setha Limsreng, Olivier Marcy, Sowath Ly, Vara Ouk, Hak Chanroeurn, Saem Thavary, Ban Boroath, Ana Canestri, Gérald Viretto, Jean-François Delfraissy, Olivier Ségéral

**Affiliations:** 1 Hôpital Calmette, Phnom Penh, Cambodia; 2 ESTHER Cambodia, Phnom Penh, Cambodia; 3 Epidemiology and Public Health Unit, Institut Pasteur in Cambodia, Phnom Penh, Cambodia; 4 ESTHER, Paris, France; 5 Internal Medecine Department, Bicêtre Hospital, le Kremlin Bicêtre, France; University of New South Wales, AUSTRALIA

## Abstract

**Background:**

Lopinavir/ritonavir (LPV/r) is widely used in Cambodia with high efficacy but scarce data exist on long-term metabolic toxicity.

**Methods:**

We carried out a cross-sectional and retrospective study evaluating metabolic disorders and cardiovascular risk in Cambodian patients on LPV/r-based antiretroviral therapy (ART) for > 1 year followed in Calmette Hospital, Phnom Penh. Data collected included cardiovascular risk factors, fasting blood lipids and glucose, and retrospective collection of bioclinical data. We estimated the 10-year risks of coronary heart disease with the Framingham, Ramathibodi-Electricity Generating Authority of Thailand (Rama-EGAT), and the Data Collection on Adverse Effects of Anti-HIV Drugs (D:A:D) risk equations. We identified patients with LDL above targets defined by the French expert group on HIV and by the HIV Medicine Association of the Infectious Disease Society of America and the Adult AIDS Clinical Trials Group (IDSA-AACTG).

**Results:**

Of 115 patients enrolled—mean age 40.9 years, 69.2% male, mean time on LPV/r 3.8 years—40 (34.8%) had hypercholesterolemia (> 2.40 g/L), and 69 (60.0%) had low HDL cholesterol (< 0.40 g/L). Twelve (10.5%), 28 (24%) and 9 (7.7%) patients had a 10-year risk of coronary heart disease ≥ 10% according to the Framingham, D:A:D, and Rama-EGAT score, respectively. Fifty one (44.4%) and 36 (31.3%) patients had not reached their LDL target according to IDSA-AACTG and French recommendations, respectively.

**Conclusion:**

Prevalence of dyslipidemia was high in this cohort of HIV-infected Cambodian patients on LPV/r. Roughly one third had high LDL levels requiring specific intervention.

## Introduction

HIV-related mortality has been dramatically reduced by the widespread use of antiretroviral treatment (ART). However, in developed countries the range of morbidity has increased, due to the emergence of cardiovascular and other non-AIDS related diseases. The role of dyslipidemia in cardiovascular morbidity is now well documented as well as the role of cardiovascular diseases as an important cause of death in HIV-infected patients [1–3]. These multifactorial complications are related to traditional risk factors including tobacco smoking, to the action of HIV itself via immune activation, as well as the toxicity of some antiretroviral drugs [4]. The role of several protease inhibitors in the occurrence of dyslipidemia is now well established and cumulative exposure to protease inhibitors has been robustly associated with a higher risk of myocardial infarction than in the general population [5].

In Cambodia, expanded access to ART has dramatically reduced morbidity associated with opportunistic infections [6], with excellent adherence and treatment outcomes [7, 8]. However, data on metabolic morbidity are limited. The expending use of ritonavir-boosted lopinavir (LPV/r) as second-line ART regimen could increase the risk of lipid disorders and contribute to an increased cardiovascular risk. Data on metabolic disorders in other South-East Asian patients on ART is scarce; high levels of dyslipidemias have been reported in Thai adults [9]. This study sought to assess the frequency of glucose and lipid metabolism disorders in Cambodian HIV-infected patients on LPV/r, and to assess their 10-year risk of developing coronary heart disease (CHD) according to the Framingham, D:A:D and Rama-Egat scoring systems [10–12].

## Methods

### Study design, settings and patients

We conducted a cross-sectional study from November, 2010 to May, 2011, in the HIV cohort of the Calmette Hospital, Phnom Penh, Cambodia. Patient follow-up and management in the cohort has been described elsewhere [7, 13]. Routine metabolic monitoring included yearly fasting lipids. Fenofibrate was available for patients with triglycerides ≥5 g/l. Patients with either virologically confirmed ART-failure or repeated toxicities were switched from non-nucleoside reverse transcriptase inhibitor (NNRTI) to protease inhibitor-containing regimen. At the time of the study, LPV/r was the only PI routinely available in Cambodia.

Patients aged ≥ 18 years were eligible for inclusion if they had been on LPV/r for ≥ 12 months at the time of assessment. We excluded patients receiving statins in the private sector.

### Study procedures and measurements

After written informed consent, patients underwent fasting lipids and glucose blood tests, complete physical examination with anthropometric measures, and a standardized questionnaire on demographic data and cardiovascular risk factors. Previous lipids and glucose measurements, CD4 count and plasma HIV RNA viral load, history of ART and other treatments were collected retrospectively from the patient's medical chart and/or the cohort database.

### Ethical considerations

The study was approved by the Cambodian National Ethics Committee for Health Research. This study was conducted in accordance with the Declaration of Helsinki [14] and all patients signed the informed consent form prior to inclusion.

### Variables

Hypercholesterolemia was defined as total cholesterol ≥ 2.4 g/l, low high-density lipoproteins cholesterol (HDL) as < 0.40 g/l, severe hypertriglyceridemia as triglycerides > 5g/l or receiving fenofibrate [15]. Elevated low-density lipoproteins cholesterol (LDL) was defined as ≥1.60 g/l, globally, and based on cardiovascular risk, individually. Hypertension was defined either as systolic blood pressure ≥140 mmHg and/or diastolic blood pressure ≥ 90 mmHg measured on both arms lying after 10-minute rest, or being on antihypertensive treatment. Diabetes mellitus was defined as either fasting glucose ≥ 1.26 g/l or being on antidiabetic treatment. Abdominal obesity was defined as waist circumference ≥ 90 cm in men and ≥80 cm in women which are measures recommended for Asian populations [16].

Individual 10 year risk of coronary heart disease (CHD) were calculated for each subject using the Framingham, D:A:D, and Rama-EGAT scoring systems, elaborated to predict the risk of angina pectoris, or myocardial infarction (MI) for the Framingham score, invasive coronary artery procedure, MI, or death from other CHD for the D:A:D score, and MI or invasive coronary artery procedure for the Rama-EGAT score [10–12] (Table 1). For the D.A.D score, the 10-year cardiovascular risk was derived from the 1-year risk score, as proposed by the authors. 10-year cardiovascular risk was considered high when ≥20%. We also evaluated the proportion of patients who had not reached LDL levels recommended by the HIV Medicine Association of the Infectious Disease Society of America and the Adult AIDS Clinical Trials Group (IDSA-AACTG) [15], and the French expert group, as well as LDL levels requiring immediate initiation of statins, according to this latter. As recommended by the HIV Medicine Association of the Infectious Disease Society of America and the Adult AIDS Clinical Trials Group, patients were classified according to their level of cardiovascular risk based on their cardiovascular history, cardiovascular risk factors, and 10 year CHD risk calculated by the Framingham equation in four groups: 1) highest risk patients with either established CHD or CHD risk equivalent defined as diabetes mellitus or 10 year risk superior to 20% according to the Framingham score; 2) patients with 2 or more cardiovascular risk factors and a 10 year CHD risk of 10 to 20%; 3) patients with 2 or more cardiovascular risk factors and a 10 year CHD risk of 0 to 10%; 4) patients with 0 or 1 cardiovascular risk factors [15]. We estimated the proportion of patients having an LDL level above or equal to the optimal target and to the treatment decision threshold recommend for their cardiovascular risk group, respectively: 1) LDL goal 1.0 g/l and treatment decision threshold 1.3 g/l; 2) LDL goal and treatment decision threshold 1.3 g/l; 3) LDL goal 1.3 g/l and treatment decision threshold 1.6 g/l; 4) LDL goal of 1.6 and treatment decision threshold of 1.9 in patients with 0 or 1 CVRF [15]. We also evaluated the proportion of patients having reached the LDL target based on the cardiovascular risk classification proposed by the French recommendations [17]: 1) LDL 1.0 in highest risk patients with either established CHD or 3 CVRF; 2) 1.3 in patients with 2 CVRF; 3) 1.6 g/l in patients with 1 CVRF; 4) 1.9 in patients with 0 CVRF.

**Table 1:** Outcomes and predictors used in the Framingham, D:A:D, and Rama-EGAT scoring systems.

	Cardiovascular risk scoring systems		
	Framingham[10]	D:A:D[11]	Rama-EGAT[12]
Outcomes	10 year risk of myocardial infarction or coronary death	10-year risk of invasive coronary artery procedure, myocardial infarction, or death from other coronary heart disease	10-year risk of invasive coronary artery procedure or myocardial infarction
Cardiovascular risk factors used as predictors			
Gender	Separate models for men and women	Male	Male
Age	Older age, discrete	Older age, continued	Older age, discrete
	Modifies risks attributed to other factors		
Total cholesterol	≥1.60 g/L	Continued	≥2.80 g/L or treatment
HDL cholesterol	<0.50 g/L; protective if ≥0.60 g/L	Continued, protective when increasing	-
Cigarette smoking	Any smoking in the past month	Current and past smoking	Current smoking
Blood Pressure/hypertension	Systolic blood pressure, discrete	Systolic blood pressure, continued	Hypertension
	Treatment for hypertension		
Abdominal obesity (circumference)	-	-	Men >90 cm, women >80 cm
Diabetes	-*	Yes	Yes
Family history	-	Coronary vascular disease	-
Exposure to antiretrovirals		Time on lopinavir or indinavirCurrently on abacavir	

HIV, human immunodeficiency virus; HDL, high-density lipoprotein cholesterol.

* Diabetes is regarded as a coronary heart disease risk equivalent but is not formally included in the 10-year risk score

Lipodystrophy was defined as the presence of at least one sign graded as moderate or severe by the investigator among all the following signs, collected independently: fat loss in face, buttocks, arms and legs for lipoatrophy; the presence of "buffalo hump"-type fat deposits in upper back, increased breast size, and abdominal obesity for lipohypertrophy.

### Statistical analyses

Comparisons between groups of patients were performed by Student t-test or non-parametric Kruskal-Wallis test for continuous variables, and Pearson chi-square or Fisher's exact test for categorical variables. Paired continuous variables were compared using Student's t test. Agreement between categorical classifications was measured by the kappa coefficient. All statistical comparisons were two-tailed, and P<0.05 was considered to be statistically significant. All analyses were performed using SAS version 11.1 (SAS Institute Inc, USA).

## Results

### Patients characteristics

**Fig 1:**
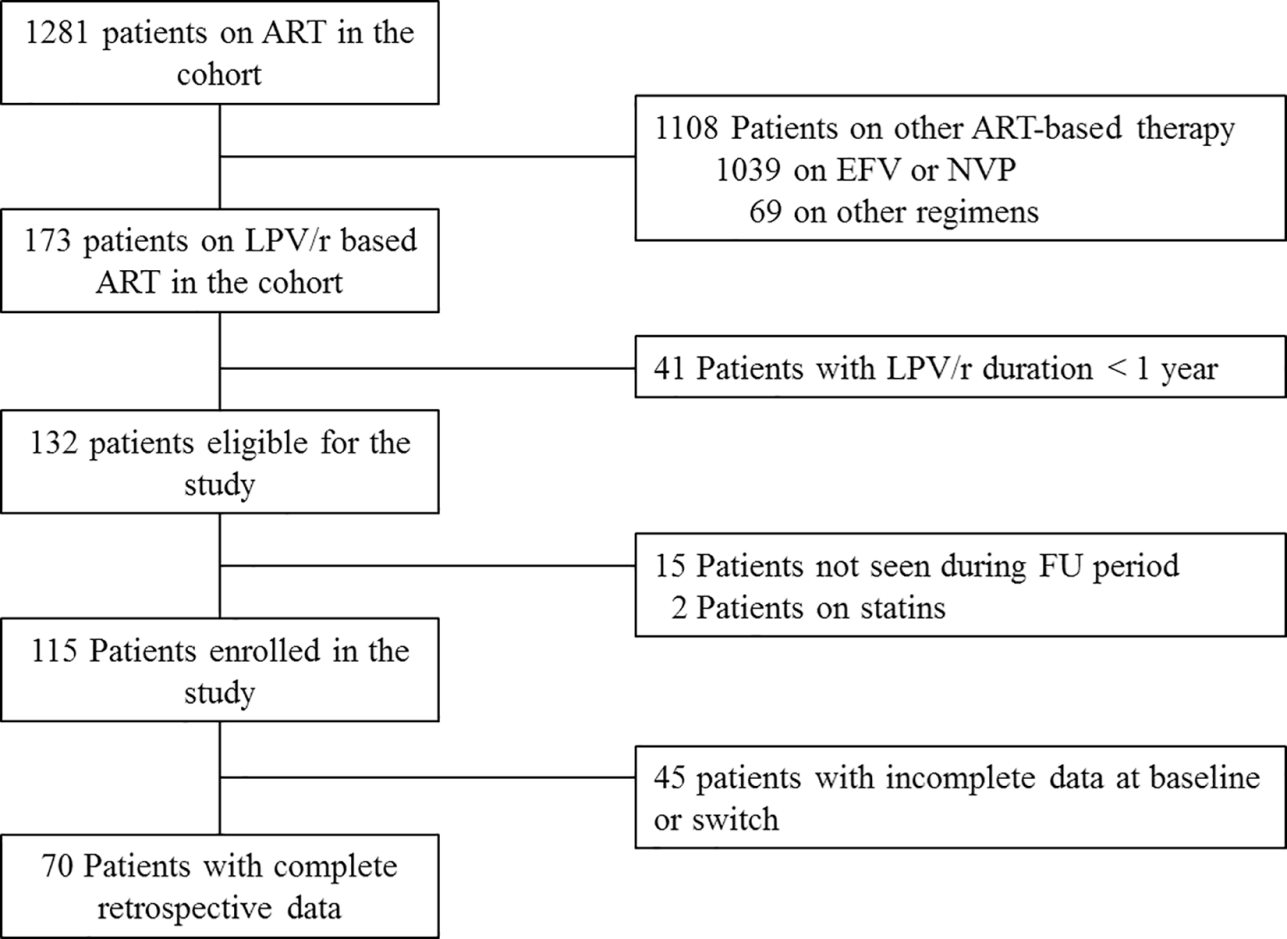
Enrolment in the study. ART: antiretroviral treatment; NNRTI: non-nucleoside reverse transcriptase inhibitors.

Of 1281 patients enrolled in the cohort, 173 (13.5%) were on LPV/r and 115 (66.5%) were enrolled in the study (Fig 1). The remaining patients were excluded because 41 had been on LPV/r ≤1 year and 15 were not seen during the study period. Additionally, 2 were excluded because they received statins.

Patients had been on LPV/r for a median time of 4.0 years (Interquartile range (IQR) 2.5–5.0) (Table 2). None reported a history of CHD, peripheral vascular disease, or cerebrovascular accident. A total of 31 patients (27.0%) were taking fenofibrate, 11 (9.6%) were on antidiabetic, and 11 (9.6%) on antihypertensive medications. Demographic and HIV-related characteristics did not differ significantly between patients on fenofibrate or not.

**Table 2:** General characteristics, dyslipidemias, cardiovascular risk factors, risk of coronary heart disease at 10 years, and Low-Density Lipoprotein cholesterol levels based on cardiovascular risk in Cambodian HIV-infected patients on Lopinavir-based ART for 1 year or more (N = 115).

	Mean ± SD or n (%)
Age	40.9 (36–44)
Gender (male)	81 (69.2)
HIV-related factors	
CD4 count (cells/μL)—median (IQR)	405 (305–537)
Time on ART (years)—median (IQR)	7.8 (6.7–8.4)
Time on LPV/r (years)—median (IQR)	4.0 (2.5–5.0)
Body mass index (kg/m^2^)	21.74 ± 3.44
Abdominal obesity*	29 (25.2)
On fenofibrate treatment	31 (27.0)
Lipid profile and dyslipidemias	
Total cholesterol (g/l)	2.28 ± 0.62
Hypercholesterolemia (> 2.40)	40 (34.8)
LDL (g/l)	1.41 ± 0.58
High LDL (> 1.60)	39 (33.9)
HDL (g/l)	0.39 ± 0.11
Low HDL (< 0.40)	69 (60.0)
Triglycerides (g/l)	2.47 ± 1.61
Triglycerides (> 5)	11 (9.6)
Severe hypertriglyceridemia (> 5 or treatment)	33 (28.7)
Major cardiovascular risk factors	
Low HDL (< 0.40 g/l)	69 (60.0)
Current smoker	10 (8.7)
Previous smoker	29 (25.2)
Age (> 45 in men or > 55 in women)^†^	20 (17.1)
Hypertension^‡^	23 (19.7)
Family history of premature CHD	0 (0.0)
Diabetes mellitus^§^	20 (17.4)
HDL ≥ 0.60 mg/l (protective factor)	6 (5.2)
Risk of coronary heart disease at 10 years	
Framingham score	
[10–20%]	11 (9.6)
> 20%	1 (0.9)
> 20% and/or diabetes mellitus (CHD risk equivalent)	21 (18.3)
D:A:D score	
[10–20%]	17 (14.5)
> 20%	11 (9.4)
Rama-Egat score	
[10–20%]	9 (7.7)
> 20%	0 (0.0)
LDL goal according to individual cardiovascular risk	
LDL goal not achieved (IDSA—AACTG)^*||*^	51 (44.4)
LDL requiring treatment (IDSA—AACTG)[15, 18]	32 (27.8)
LDL goal not achieved (French recommendations)	36 (31.3)

SD, standard deviation; HIV, human immunodeficiency virus; ART, antiretroviral treatment, LPV/r, ritonavir boosted lopinavir; LDL, low-density lipoprotein cholesterol; HDL, high-density lipoprotein cholesterol; CHD, coronary heart disease.

* waist circumference ≥ 90 cm in men and ≥80 cm in women

† in French recommendation, age is considered a risk factor ≥50 in men and ≥60 in women (n = 9, 7.8%)

‡ Defined either as systolic blood pressure ≥140 mmHg and/or diastolic blood pressure ≥ 90 mmHg or being on antihypertensive treatment

§ Defined as either fasting glucose ≥ 1.26 g/l or being on antidiabetic treatment.

### Dyslipidemias on lopinavir/ritonavir

Lipid profiles and prevalence of dyslipidemia are displayed in Table 2. Forty (34.8%) patients had hypercholesterolemia, 39 (33.9%) had high LDL, 69 (60.0%) had low HDL, and 33 (28.7%) had severe hypertriglyceridemia (> 5 g/L). Patients on fenofibrate had higher total cholesterol (2.56 ± 0.85 Vs. 2.18 ± 0.48, p 0.0027), lower HDL (0.34 ± 0.10 Vs. 0.41 ± 0.11, p 0.0012), and higher triglycerides (4.01 ± 2.31 Vs. 1.95 ± 1.07 p<0.0001). LDL did not differ between patients on fenofibrate and untreated patients (1.47 ± 0.80 Vs. 1.39 ± 0.47, p 0.4611).

At ART initiation, 58 (54.2%) patients out of 105 with HDL available had a low HDL. Only 70 patients had lipid levels available at ART initiation and ART switch from NNRTI to LPV/r based ART. LDL levels were measurable in 64 at ART initiation and 60 at ART switch. Comparisons between lipid levels at ART initiation and evaluation on LPV/r and between ART switch and evaluation on LPV/r are displayed in Table 3. Globally, from ART initiation to evaluation, total cholesterol increased by 0.60 ± 0.61 g/l, and LDL increased by 0.51 ± 0.62. On LPV/r, total cholesterol increased by 0.46 ± 0.68 g/l, LDL increased by 0.48 ± 0.64 g/l, and HDL decreased by 0.13 ± 0.41 g/l. In the 84 patients without fenofibrate, total cholesterol and LDL were increased compared to ART initiation levels (p <0.0001 and p <0.0001); however HDL and triglycerides were unchanged (p = 0.22 and p = 0.44).

**Table 3:** Evolution of lipid levels between ART-initiation of antiretroviral treatment, switch to lopinavir/ritonavir and evaluation (N = 70).

	ART initiation	Switch to LPV/r	On LPV/r	P Value	P Value
	(1)	(2)	(3)	(1) Vs. (3)	(2) Vs. (3)
	Mean ± SD	Mean ± SD	Mean ± SD		
Total cholesterol (g/L)	1.67 ± 0.56	1.80 ± 0.69	2.27 ± 0.71	**<0.0001**	**<0.0001**
LDL cholesterol (g/L)	*0.85 ± 0.47	^†^0.87 ± 0.45	*1.37 ± 0.47	**<0.0001**	**<0.0001**
HDL cholesterol (g/L)	0.48 ± 0.41	0.52 ± 0.39	0.39 ± 0.11	**0.0083**	0.0742
Triglycerides (g/L)	2.16 ± 1.92	2.58 ± 3.19	2.48 ± 1.54	0.7951	0.0990

ART, antiretroviral treatment; LPV/r, lopinavir/ritonavir; SD, standard deviation; LDL, low density lipoprotein cholesterol; HDL, high density lipoprotein cholesterol.

* N = 64

^†^N = 60

### Cardiovascular risk and target LDL levels

Major cardiovascular risk factors are displayed in Table 2. When considering all risk factors exclusive of LDL, the median number of risk factor per patient was 1 (IQR: 0 to 2). 47 patients had only risk factor and 38 of them (80.9%) had isolated low HDL.

When assessing 10-year risk of CHD, 1 (0.9%), 11 (9.6%) and 0 (0%) patients were considered at high CHD risk by the Framingham, D:A:D and Rama-EGAT score, respectively. Additionally, following US recommendations, 20 patients with diabetes should be considered at high cardiovascular risk, raising the number to 21 (18.3%).

Twelve (10.5%), 28 (24%) and 9 (7.7%) patients were considered at intermediate or high 10-year risk of CHD by the Framingham, D:A:D, and Rama-EGAT score, respectively (Table 2). Individual characteristics of the patients considered at intermediate or high 10-year risk of CHD are showed on Table 4.

**Table 4:** Individual characteristics of patients at intermediate or high cardiovascular risk.

#	Age (y)	Sex	Time on (y)		Ongoing ART	CD4 (/mm3)	Smoker	Body Weight (kg)	Height (cm)	Abdo. Circum. (cm)	SBP	DBP	Treatment			Fasting blood lipids and glucose (g/L)					Cardiovascular risk scores			LDL target reached	
			ART	LPV							(mm Hg)		HTN	DM	TG	Gly	Chol	HDL	LDL	TG	Framingham	Rama-Egat	DAD	US	FR
2	42	M	5.3	4.2	DDI 3TC LPV	576	Current	51	160	72	120	80	0	0	0	2.45	1.29	0.31	0.73	1.27	3%	8%	14.7%	1	1
3	42	M	7.4	4.4	TDF 3TC LPV	470	Current	73	169	86	115	83	0	0	1	0.96	4.33	0.59	3.28	2.8	12%	3%	27.1%	0	0
7	49	M	7.3	4.8	DDI 3TC LPV	346	Current	84	170	100	108	65	0	0	0	1.27	2.59	0.46	1.77	1.9	20%	20%	35.5%	0	0
9	48	M	7.4	4.9	AZT 3TC LPV	537	Current	69	168	90	120	76	0	0	0	1.01	2.2	0.25	0.95	6.25	20%	4%	21.2%	1	1
11	81	M	3.7	1.4	TDF 3TC LPV	433	Past	44	164	72	125	62	0	0	0	1.07	2.26	0.38	1.58	1.53	20%	14%	59.4%	0	0
13	45	M	8.4	1.4	TDF 3TC LPV	425	Past	72	166	90	126	74	0	0	0	1	3.02	0.47	2.21	1.74	10%	3%	10.0%	0	0
15	62	M	12.4	9.5	TDF EFV LPV	455	No	66	170	89	143	57	1	0	0	3.39	2.54	0.77	1.63	0.71	12%	20%	49.1%	0	0
16	43	M	10.7	5.8	DDI 3TC LPV	91	Current	57	174	77	100	70	0	0	0	0.96	2.32	0.4	1.64	1.4	8%	3%	13.7%	0	0
20	37	M	4.2	3.4	ABC 3TC LPV	513	No	56	166	78			1	1	1	0.9	4.85	0.27	3.54	5.2	8%	6%	41.8%	0	0
23	37	M	8.4	4.9	DDI 3TC LPV	560	Current	56	159	81	120	80	0	0	0	0.8	2.99	0.23	1.57	5.97	30%	2%	16.5%	0	0
28	32	F	8.4	3.0	TDF 3TC LPV	603	No	65	162	92	120	80	0	1	0	1.35	1.76	0.33	1.21	1.11	0%	4%	1.9%	0	1
31	43	M	7.4	3.3	TDF ABC LPV	190	Past	42	163	71	110	70	0	1	0	0.86	2.65	0.36	1.93	1.79	4%	5%	26.8%	0	0
34	43	M	3.8	3.3	AZT 3TC LPV	621	Current	52	168	79	110	80	0	0	0	1.1	1.9	0.29	0.79	3.61	6%	3%	10.9%	1	1
38	49	F	8.5	2.3	DDI 3TC LPV	349	No	44	158	81	116	78	1	1	0	4.44	2.51	0.38	1.7	1.68	2%	8%	7.8%	0	0
42	62	M	8.3	5.0	DDI 3TC LPV	792	Current	63	168	82	127	78	0	0	1	1.04	2	0.31	1.31	1.91	16%	14%	41.0%	0	0
50	40	M	9.7	2.8	DDI 3TC LPV	320	No	60	164	81	108	71	0	0	1	1.34	3.91	0.26	2.62	5.12	6%	5%	18.4%	0	0
51	36	M	10.5	2.9	ABC 3TC LPV	244	No	69	173	82	145	82	0	0	1	1.12	3.87	0.35	2.82	3.5	6%	2%	12.9%	0	0
52	38	M	8.3	2.7	DDI 3TC LPV	308	No	57	165	76	120	82	0	0	0	1.26	2.67	0.39	1.8	2.42	3%	4%	7.3%	0	0
55	40	M	5.5	3.7	TDF 3TC LPV	104	No	72	175	88	123	78	0	1	1	0.92	1.82	0.3	0.35	7.82	2%	5%	6.4%	1	1
57	51	M	13.6	4.4	DDI 3TC LPV	424	No	59	168	83	149	80	1	1	1	1.48	1.81	0.18	0.26	6.83	10%	20%	17.3%	1	1
59	50	M	8.3	2.0	ABC 3TC LPV	415	No	62	170	86	113	86	0	0	0	1.01	2.34	0.38	1.3	3.32	8%	4%	11.4%	0	1
61	46	F	3.5	1.2	3TC SQV LPV	141	No	46	155	78	107	76	0	1	1	1.54	3.88	0.38	2.3	6.2	3%	4%	12.1%	0	0
68	51	M	8.4	4.8	DDI 3TC LPV	562	Past	62	170	84	100	70	1	1	1	1.12	2.67	0.6	1.63	2.2	5%	20%	21.9%	0	0
73	46	M	8.2	3.9	DDI 3TC LPV	512	Past	57	168	75	129	68	0	0	0	0.94	2.27	0.59	1.37	1.59	4%	3%	12.3%	1	1
74	44	M	7.4	1.1	TDF ABC LPV	368	Past	57	165	84	0		0	0	0	0.97	2.15	0.41	1.26	2.37	2%	4%	10.5%	1	1
76	66	M	8.5	3.5	DDI 3TC LPV	362	Past	70	168	91	134	91	0	0	0	0.9	2.84	0.52	1.88	2.2	20%	20%	36.2%	0	0
77	42	M	7.8	5.0	DDI 3TC LPV	463	Past	77	175	90	131	76	0	0	0	1.01	2.97	0.5	2.15	1.63	5%	2%	10.1%	0	0
80	57	M	11.5	3.9	ABC 3TC LPV	342	No	47	167	76	133	70	0	0	1	0.92	1.99	0.36	1.07	2.8	12%	6%	18.4%	1	1
86	45	M	7.8	5.3	DDI 3TC LPV	304	Past	57	160	88	98	66	1	1	1	1.25	2.16	0.23	1.03	4.52	6%	14%	18.7%	0	0
99	44	M	11.7	2.8	DDI 3TC LPV	280	Past	59	165	80	110	82	0	0	0	1.33	2.25	0.28	1.52	4.27	3%	5%	15.2%	0	0
100	47	M	9.6	2.4	DDI 3TC LPV	289	Past	60	164	86	147	89	0	0	0	1.05	3.2	0.39	2.17	3.19	16%	5%	15.8%	0	0
101	49	M	7.0	5.0	DDI 3TC LPV	535	Past	61	160	86	146	70	0	0	0	1.85	2.24	0.43	1.58	1.15	6%	14%	22.3%	0	1
103	32	M	7.6	2.4	DDI 3TC LPV	616	No	58	160	80	122	86	0	0	1	1.5	3.06	0.38	1.5	6.81	1%	4%	6.1%	0	0
109	43	M	7.7	2.7	DDI 3TC LPV	359	No	78	165	95	117	74	0	1	1	1.19	1.97	0.28	0.73	4.81	2%	9%	7.9%	1	1
111	43	F	7.8	1.1	TDF DDI LPV	261	No	51	153	76	117	84	0	0	0	1.3	1.92	0.34	1.26	2.1	0%	3%	3.7%	0	1
116	40	M	7.8	3.6	DDI 3TC LPV	316	No	73	167	90	127	80	0	1	1	1.28	2.4	0.3	1.22	4.38	4%	5%	8.9%	0	1
121	53	M	8.8	5.4	TDF ABC LPV	408	Past	63	170	88	134	82	0	0	0	1.22	1.87	0.54	1.11	0.84	5%	4%	18.6%	1	1

ART, antiretroviral treatment; LPV, lopinavir(/ritonavir); SBP, systolic lood pressure; DBP, diastolic blood pressure; HTN, hypertension; DM, diabetes mellitus; TG, triglycerides; Chol, total cholesterol; Glu, glucose; HDL, high-density lipoprotein cholesterol; LDL, low-density lipoprotein cholesterol; DDI, didanosine; 3TC, lamivudine; TDF, tenofovir; AZT, zidovudine; 3TC, lamivudine; ABC, abacavir; SQV, saquinavir.

Fifty one (44.4%) and 36 (31.3%) patients had not reached their LDL target according to IDSA-AACTG and French recommendations, respectively (87.0% agreement, Kappa 0.73, SE 0.09, p<0.0001) (Table 5). Furthermore, 32 (27.8%) patients had LDL level requiring immediate treatment according to IDSA-AACTG (94.8% agreement with French target level, Kappa: 0.87, SE 0.09, p<0.0001). Of the 38 patients with isolated low HDL, 10 (26.3%) had not reached their LDL target according to IDSA–AACTG recommendations.

**Table 5:** Agreement between French recommendations and IDSA AACTG recommendations regarding achievement of / treatment decision based on LDL goal related to CV risk level.

	LDL goal based on CV risk level (French recommendations)		Kappa (SD)
	Not reached	Reached	
	(n = 36)	(n = 79)	
LDL treatment recommendation based on CV risk level (IDSA AACTG)			0.87 (0.09)
Treatment	31	1	
No treatment	5	78	
LDL goal based on CV risk level (IDSA AACTG)			0.72 (0.09)
Not reached	36	15	
Reached	0	64	

LDL, low-density lipoprotein cholesterol; CV, cardiovascular; SD, standard deviation; IDSA AACTG, Infectious Disease Society of America and the Adult AIDS Clinical Trials Group.

## Discussion

Prevalence of dyslipidemias was high in this cohort of HIV-infected Cambodian patients on LPV/r based-regimen for a median duration of 4 years. Up to 42% of the patients were considered as having LDL higher than desired and 30% need immediate intervention including dietetic rules and treatment by statins.

Our findings were consistent with a previous report in Cambodia showing an overall prevalence of dyslipidemias of 73% after 4 years on ART, in a cohort with 10% of patients on LPV/r based ART. Total cholesterol is particularly high in our patients compared to other cohorts of HIV-infected patients on LPV/r [2, 19] but consistent with early report from the French APROCO cohort of patients on protease inhibitor-based ART before use of ritonavir boosting [20]. A more recent study in 170 Thai patients on PI-based ART reported a mean total cholesterol of 2.6 g/L and worsened lipid profiles anomalies in patients on ritonavir-boosted PI regimens [9]. Analysis of data collected routinely in cohort follow-up showed that total cholesterol, triglycerides, and LDL levels increased on LPV/r, a well known effect of this therapeutic regimen [19, 21, 22]. Our study, with 3 time points confirmed these results in a South-East Asian population.

HDL levels were low in our patients at ART initiation, which was expected, low HDL being a common HIV-induced dyslipidemia and possibly a main contributor to CHD risk in HIV-infected population [23]. Low HDL levels have been described in ART-naïve HIV-infected individuals in caucasian and other populations [24–26]. Surprisingly, in our patients, HDL levels decreased on LPV/r whereas other studies reported an increase on ART [27]. This is of particular concern, even in the absence of other cardiovascular risk factors, as isolated low HDL has been shown to be associated to elevated cardiovascular risk in various studies [28] and specifically in Asian populations [29].

Hypercholesterolemia was 5 to 10-fold higher than estimated in a recent survey in the Cambodia general population which showed a global and urban hypercholesterolemia prevalence of 3.2% and 7%, respectively, using similar threshold or the presence of lipid lowering treatments [30]. Considering other risk-factors, reported tobacco smoking was much lower in our study compared to the general population survey which reported a 49.3% and 4.8% current smoking rate in men and women, and prevalence of hypertension and diabetes in the much higher than the 3.1 and 3.2% found in the survey, respectively. The prevalence observed in our study may have been overestimated due to the cross-sectional design, but the frequency of diabetes or antihypertensive medications intake confirmed the importance of these disorders.

The RAMA-EGAT score, specifically designed for a South-East Asian population, showed that cardiovascular risk was higher in this study than reported previously in Thai patients of similar age and duration on ART, including 60% on protease inhibitor-based ART [12]. Score performance for the prediction of 10-year cardiovascular risk differed from the study in Thailand which showed a good agreement between the D:A:D and the RAMA-EGAT scores, where in our study D:A:D predicted a higher cardiovascular risk in patients while Framingham and RAMA-EGAT scores had much lower estimates, which may be due to the high prevalence of diabetes.

The IDSA-AACTG and French scores agreed to identify roughly 1/3 of patients needing immediate treatment for LDL levels above the desired target. However statins are expensive and not widely available in setting such as Cambodia. Diet, lifestyle changes, and change of ART if 10-year CV risk > 20% seem to be the most appropriate solution [31]. Atazanavir, a newer protease inhibitor associated with only modest changes in lipid profile [32], is also becoming increasingly available in resource limited-setting and is recommended as first choice for second-line regimen since 2012 in Cambodia.

There are limitations to this study. First, patients from this single site may not be representative of the general HIV-infected population in Cambodia. As shown in other studies in Asia, lipid levels and cardiovascular risk in urban population may be different from rural population [33, 34]. We may also have overestimated hypertension and diabetes due to the essentially cross-sectional design of our study. Second, 27% of patients were on fenofibrate which may have led to decreased LDL and increased HDL levels. However the poor control of lipids emphasizes the interest for a better management of metabolic disorders in these patients. At last, none of the risk equations used in our study were validated specifically in HIV-infected South-East Asian populations. More epidemiological studies are needed to document incidence of cardio-vascular and other non-AIDS related morbidity in HIV-infected populations in South-East Asia and Africa. This would help validating risk equations and decision-making on risk reduction at individual level.

Dyslipidemias were frequent in HIV-infected Cambodian patients on LPV/r for more than one year, contributing to an elevated cardiovascular risk. The particularly high prevalence of low HDL warrants further investigation and better evaluation of cardiovascular risk in Asian population. In Cambodia, the progressive switch from LPV/r to atazanavir will certainly contribute to an improvement in lipid profiles and will need further evaluation. However, this study highlights the necessity to better take into account non-AIDS related co-morbidities in limited-resources settings as HIV care becomes chronic care and as these countries will face major challenges on management of cardiovascular morbidity in general [35].

## Supporting Information

S1 Data This is the S1 Data, which correspond to the entire data’s table of the study. (XLS)
